# Sampling Method Affects HR-MAS NMR Spectra of Healthy Caprine Brain Biopsies

**DOI:** 10.3390/metabo11010038

**Published:** 2021-01-06

**Authors:** Annakatrin Häni, Gaëlle Diserens, Anna Oevermann, Peter Vermathen, Christina Precht

**Affiliations:** 1 Clinical Radiology, Department of Clinical Veterinary Medicine, Vetsuisse Faculty, University of Bern, Länggassstr. 124, 3012 Bern, Switzerland; anhaeni@bluewin.ch; 2 Departments of BioMedical Research and Radiology, University and Inselspital Bern, sitem-insel AG, Freiburgstr. 3, 3010 Bern, Switzerland; gaelle.diserens@hotmail.com (G.D.); peter.vermathen@insel.ch (P.V.); 3 NeuroCenter, Division of Neurological Sciences, Department of Clinical Research and Veterinary Public Health, Vetsuisse Faculty, University of Bern, Bremgartenstr. 109a, 3012 Bern, Switzerland; anna.oevermann@vetsuisse.unibe.ch

**Keywords:** stereotactic brain biopsy, postmortem brain sampling, metabolism, ischemia, goat

## Abstract

The metabolic profiling of tissue biopsies using high-resolution–magic angle spinning (HR-MAS) ^1^H nuclear magnetic resonance (NMR) spectroscopy may be influenced by experimental factors such as the sampling method. Therefore, we compared the effects of two different sampling methods on the metabolome of brain tissue obtained from the brainstem and thalamus of healthy goats by ^1^H HR-MAS NMR spectroscopy—in vivo-harvested biopsy by a minimally invasive stereotactic approach compared with postmortem-harvested sample by dissection with a scalpel. Lactate and creatine were elevated, and choline-containing compounds were altered in the postmortem compared to the in vivo-harvested samples, demonstrating rapid changes most likely due to sample ischemia. In addition, in the brainstem samples acetate and inositols, and in the thalamus samples ƴ-aminobutyric acid, were relatively increased postmortem, demonstrating regional differences in tissue degradation. In conclusion, in vivo-harvested brain biopsies show different metabolic alterations compared to postmortem-harvested samples, reflecting less tissue degradation. Sampling method and brain region should be taken into account in the analysis of metabolic profiles. To be as close as possible to the actual situation in the living individual, it is desirable to use brain samples obtained by stereotactic biopsy whenever possible.

## 1. Introduction

Various studies of the metabolic profiling of neoplastic and non-neoplastic brain diseases [1] using high-resolution–magic angle spinning (HR-MAS) ^1^H nuclear magnetic resonance (NMR) spectroscopy of tissue samples have shown the potential of this method as a diagnostic and prognostic tool [2,3]. Metabolic profiles can complement histopathological examination [4], and provide new information for tumor classification and grading [2,3]. Some metabolites may be used as biomarkers and thus improve brain tumor diagnosis [5].

A metabolic profile should represent the situation in a patient’s body as accurately as possible, and alterations in the biochemical profile due to experimental manipulation may introduce bias, which may lead to misinterpretation. Enzymatic changes can vary due to experimental factors, including but not limited to factors before (e.g., anesthesia, euthanasia), during (e.g., sampling method) and after sampling (e.g., processing, storage), and NMR experimental factors, and they appear in all cases until samples are frozen or fixed [1,6].

A number of studies in laboratory rodents have examined the influences of different experimental factors on brain metabolites, either using intact tissue biopsies [6,7] or tissue extracts [8,9,10,11,12]. All samples were taken after the animals had been killed by liquid funnel-freezing [6,10,11], by cervical dislocation [7,12] or decapitation [9,10], by intravenous administration of potassium chloride [8,11] or by asphyxiation [11], and demonstrate that various factors may influence the peri- and postmortem metabolic profile. However, it is desirable to evaluate the metabolic changes that occur in a situation representing the clinical routine in human medicine and in larger mammals, where brain tissue samples for HR-MAS examination can either be taken after the surgical removal of a brain mass or by stereotactic brain biopsy under general anesthesia. The period of sample ischemia due to the time delay before the freezing of the sample varies between a few minutes, in the case of stereotactic biopsy, and several minutes to hours in the case of surgical removal, depending on the complexity and duration of the surgical procedure. The surgical removal of a brain mass also involves the cauterization of large blood vessels that supply the mass and may lead to prolonged intraoperative ischemia of the sample [6].

Taking biopsies using a minimally invasive stereotactic approach in vivo under general anesthesia results in shorter time periods until the processing or freezing of the sample compared to surgical biopsies. Consequently, we hypothesize that there will be fewer signs of tissue degradation in stereotactic biopsies.

The aim of this study was to investigate the effect of the sampling method on healthy brain tissue. We compared the HR-MAS spectra of brain biopsies from four healthy goats harvested stereotactically “in vivo” under general anesthesia with samples harvested surgically “postmortem” shortly after euthanasia of the same four healthy goats. The postmortem harvested samples with an ischemia time of 20 min are a surrogate to the situation of surgical brain mass excision in human medicine.

## 2. Results

**Figure 1:**
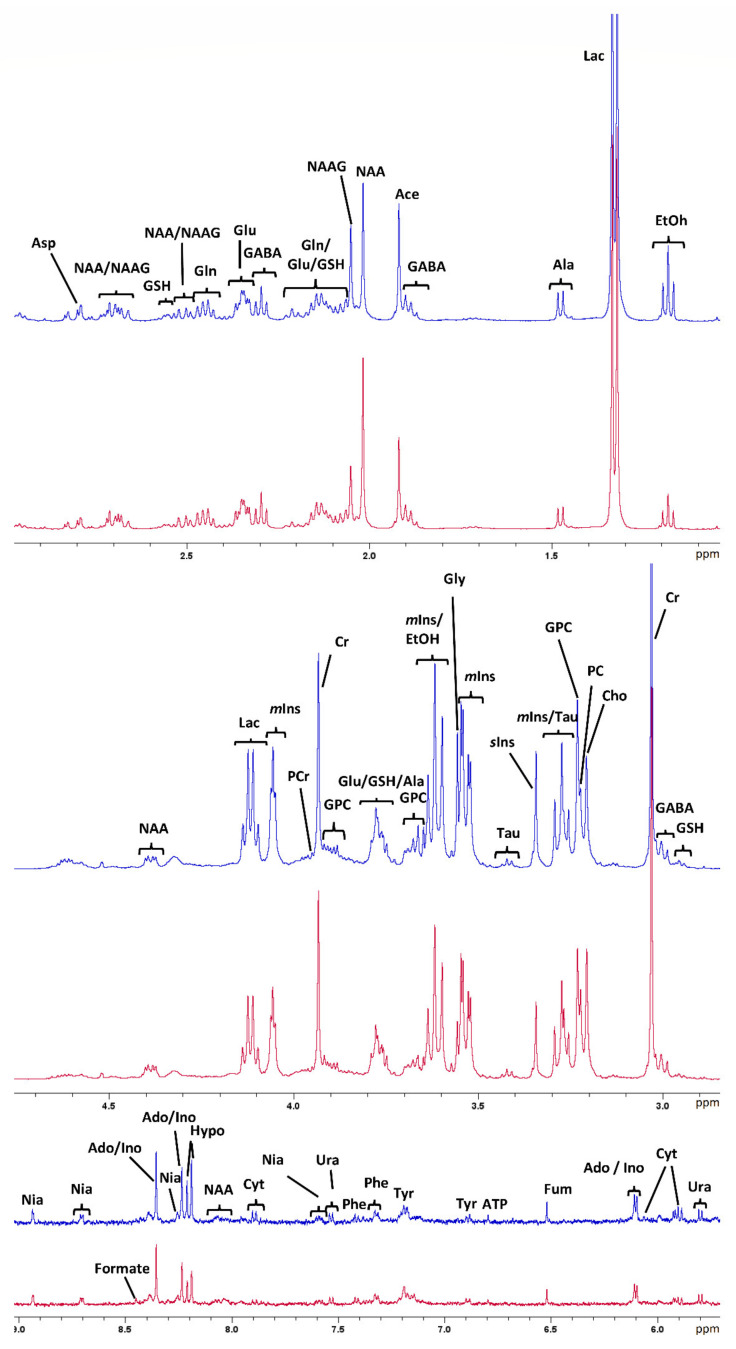
Assigned one-dimensional sum spectra (between 0.5 and 9 ppm) of all in vivo- and postmortem-harvested samples of the thalamus (red) and the brainstem (blue), respectively. Aromatic region (5.5–9 ppm) scaled up by a factor of 4. Ace, acetate; Ado, adenosine; Ala, alanine; Asp, aspartate; ATP, adenosine triphosphate; Cho, choline; Cr, creatine; Cyt, cytidine; EtOH, ethanol; Fum, fumarate; GABA, ɣ-aminobutyric acid; Gln, glutamine; GSH, glutathione; Glu, glutamate; Gly, glycine; GPC, glycerophosphocholine; Hypo, hypoxanthine; Ino, inosine; Lac, lactate; *m*Ins, *myo*-inositol; NAA, *N*-acetylaspartate; NAAG, *N*-acetylaspartylglutamate; Nia, Niacin; PC, phosphocholine; PCr, phosphocreatine; Phe, phenylalanine *s*Ins, *scyllo*-inositol; Tau, taurine; Tyr, tyrosine; Ura, uracil.

One in vivo-harvested biopsy of the brainstem was excluded due to poor spectral quality. Thus, 31 spectra were statistically evaluated. The in vivo-harvested biopsies were lighter (mean 7.11 mg; range 2.90–11.7 mg) than the postmortem-harvested samples (mean 9.54 mg, range 7.20–11.60 mg). One-dimensional sum spectra of all the in vivo- and postmortem-harvested samples of the brainstem and thalamus, respectively, are shown in Figure 1, together with spectral assignments.

### 2.1. Metabolic Profiles of In Vivo and Postmortem Samples of the Brainstem and Thalamus with Regard to the Individual Animal

As two samples of both the brain regions of each individual goat (one sample from the right side and one sample from the left side) were used for this study, the samples were also investigated to reveal the possible influence of their origin in the individual animal as a confounding factor. The principal component analysis (PCA) of the in vivo- and postmortem-harvested samples did not show a clustering of the samples obtained in vivo from the thalamus and brainstem of individual animals, as shown in Figure S1a. Similarly, only a vague clustering of the samples from individual animals is observed in the PCA of the postmortem-harvested samples from the brainstem and thalamus, as shown in Figure S1b. In partial least squares–discriminant analysis (PLS-DA) using the animal as the Y-variable of in vivo brainstem samples, postmortem brainstem samples, in vivo thalamus samples and postmortem thalamus samples, a clustering of the samples obtained from individual animals is present, as shown in Figure S2. Although the PLS-DA results may indicate that the origin from an individual animal has an influence on the metabolome, in the sense of the lower intra-individual than inter-individual variability, it seems reasonable in view of the unsupervised PCA results to use both samples of each individual animal for the statistical evaluation of the influence of the sampling method.

### 2.2. Metabolic Profiles of In Vivo and Postmortem Samples with Regard to the Brain Region (Brainstem and Thalamus)

A chemometric analysis of the samples was also performed in order to determine the possible differences in the metabolic profile of the two different brain region samples: brainstem and thalamus. The analysis for differences in the metabolic profiles of the brainstem and thalamus showed a clear separation in the PLS-DA for biopsies harvested both in vivo (Figure S3) and postmortem (Figure S4).

The important discriminating metabolites were *N*-acetylaspartate (NAA) and *N*-acetylaspartylglutamate (NAAG); in both the in vivo- and postmortem-harvested samples, NAA was higher in the thalamus and NAAG in the brainstem. In both the in vivo- and postmortem-harvested samples, phosphocholine (PC) was increased in the thalamus compared to the brainstem. Only in the in vivo-harvested biopsies, but not in the postmortem-harvested biopsies, other metabolites of the Choline group were also important discriminators; choline (Cho) was increased in the thalamus samples and glycerophosphocholine (GPC) was increased in the brainstem samples. Other metabolites, which were important discriminators and showed similar patterns in both the in vivo- and postmortem-harvested samples, were Glycine (Gly) and *myo*-inositol (*m*Ins), which both showed a relative increase in the brainstem compared to the thalamus.

ɣ-aminobutyric acid (GABA) was relatively decreased in the thalamus compared to the brainstem in in vivo-harvested biopsies, but was relatively increased in the thalamus compared to the brainstem biopsies in postmortem-harvested biopsies.

As the metabolic profiles of the brain regions were clearly different, datasets of the brainstem and thalamus samples were analyzed separately with regard to the influence of the sampling method.

### 2.3. Metabolic Profiles of the Brainstem and Thalamus with Regard to the Sampling Method (In Vivo and Postmortem)

**Figure 2:**
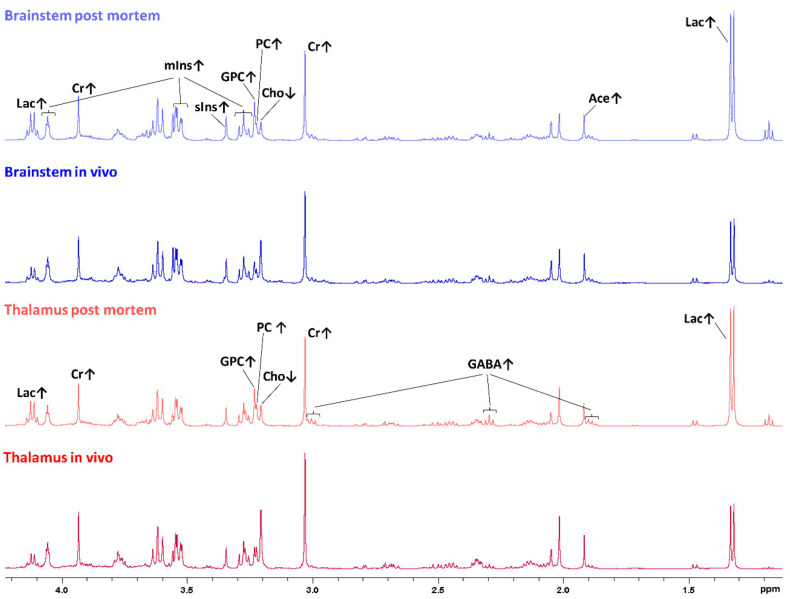
One-dimensional sumspectra (between 0.5 and 4.5 ppm, scaled relative to the total intensity between 0.5 and 4.5 ppm) of postmortem brainstem samples (light blue), in vivo brainstem samples (dark blue), postmortem thalamus samples (light red) and in vivo thalamus samples (dark red). Discriminating metabolites between postmortem and in vivo samples of the brainstem or thalamus, respectively, are assigned, and relatively increased or decreased concentrations are indicated by arrows pointing up or down, respectively. Ace, acetate; Cho, choline; Cr, creatine; GABA, ɣ-aminobutyric acid; GPC, glycerophosphocholine; Lac, lactate; *m*Ins, *myo*-inositol; PC, phosphocholine; *s*Ins, *scyllo*-inositol.

One-dimensional mean spectra of postmortem brainstem samples, in vivo brainstem samples, postmortem thalamus samples and in vivo thalamus samples are shown in Figure 2, highlighting the metabolic differences observed in PLS-DA, as described below.

**Figure 3:**
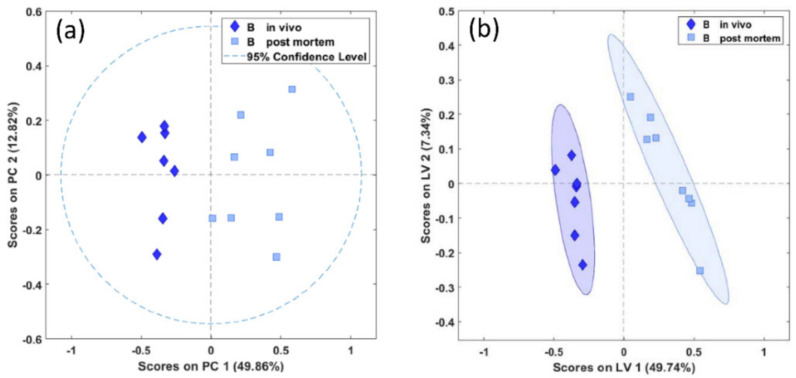
PCA (**a**) and PLS-DA (**b**) scores plots of in vivo (diamonds) and postmortem (squares) biopsies of the brainstem demonstrating significant separation between the biopsy types (PCA and PLS-DA, *p* = 0.001). PC, principal component in arbitrary units; LV, latent variable in arbitrary units.

A significant separation between in vivo- and postmortem-harvested biopsies of the brainstem was achieved both with the unsupervised PCA (Wilcoxon, *p* < 0.01) and with the supervised PLS-DA (Wilcoxon, *p* = 0.001), as shown in Figure 3a,b.

**Figure 4:**
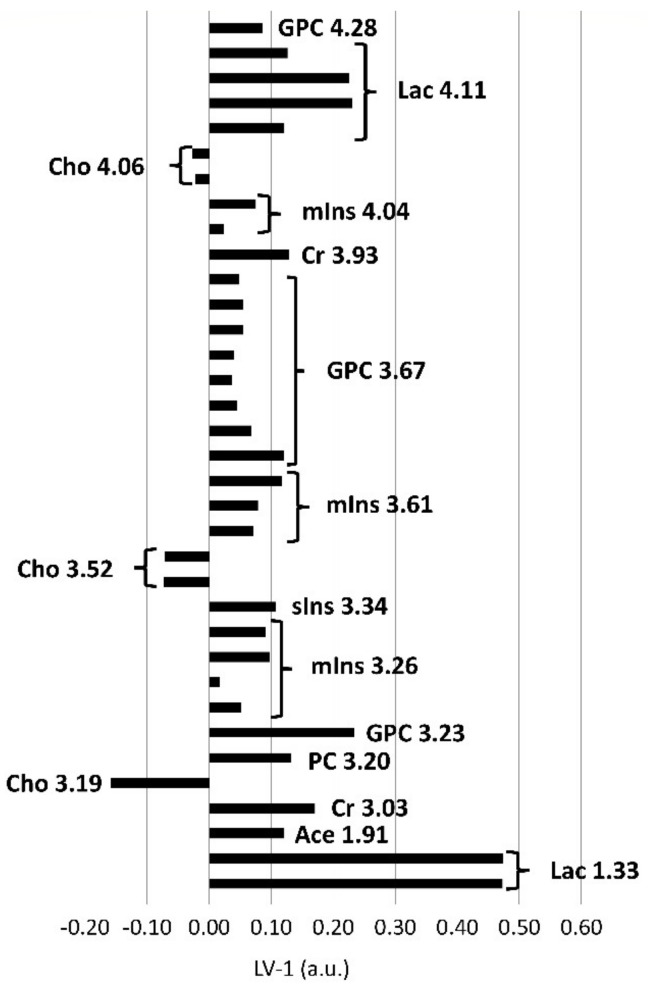
PLS-DA loadings of the first PLS-DA component (LV-1, arbitrary units) of brainstem biopsies demonstrating significant differences between in vivo and postmortem biopsies. Positive loadings correspond with metabolites present in increased concentrations, negative loadings with decreased concentrations in the in vivo biopsies compared to the postmortem biopsies. The length of the bar is positively correlated with the importance as a discriminator between the groups in the PLS-DA model. Excerpt of buckets assigned to metabolites and their center frequencies in ppm beyond a PLS-DA coefficient of +/−0.1, below or above which they were considered as important discriminators. Additionally, corresponding resonances from the same metabolites including resonances below +/−0.1 are also shown. Ace, acetate; Cho, choline; Cr, creatine; GPC, glycerophosphocholine; PC, phospchocholine; Lac, lactate; *m*Ins, *myo*-inositol; *s*Ins, *scyllo*-inositol.

In the postmortem-harvested brainstem samples biopsies, Cho was decreased and lactate (Lac), creatine (Cr), PC, GPC, *m*Ins, *scyllo*-inositol (*s*Ins) and acetate (Ace) were increased compared with the in vivo-harvested biopsies (Figure 4). Other metabolites, such as alanine (Ala), aspartate (Asp), glutamine (Gln), glutamate (Glu), glutathione (GSH), Gly, NAAG, NAA, Phosphocreatine (PCr), and Taurine (Tau), did not exceed the arbitrarily chosen PLS-DA coefficient threshold value of +/−0.1.

**Figure 5:**
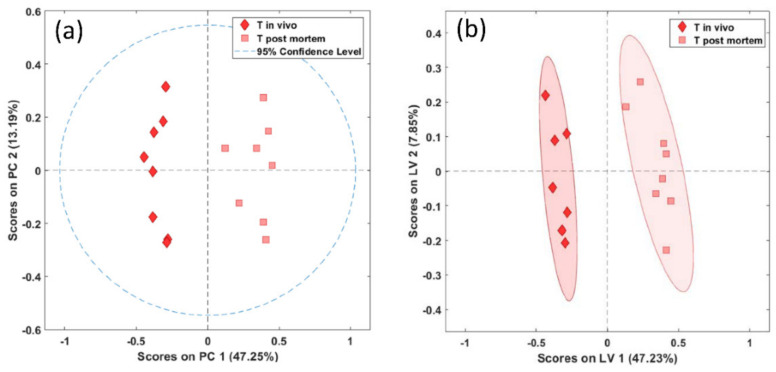
PCA (**a**) and PLS-DA (**b**) score plots of in vivo (diamonds) and postmortem (squares) biopsies of the thalamus demonstrating significant separation between the biopsy types (PCA, *p* = 0.001, PLS-DA, *p* = 0.004).

Similarly to the brainstem, in the biopsies of the thalamus we found a significant difference between the in vivo- and postmortem-harvested samples, both with unsupervised PCA (Wilcoxon, *p* < 0.01) and supervised PLS-DA (Wilcoxon, *p* = 0.004) (Figure 5a,b).

**Figure 6:**
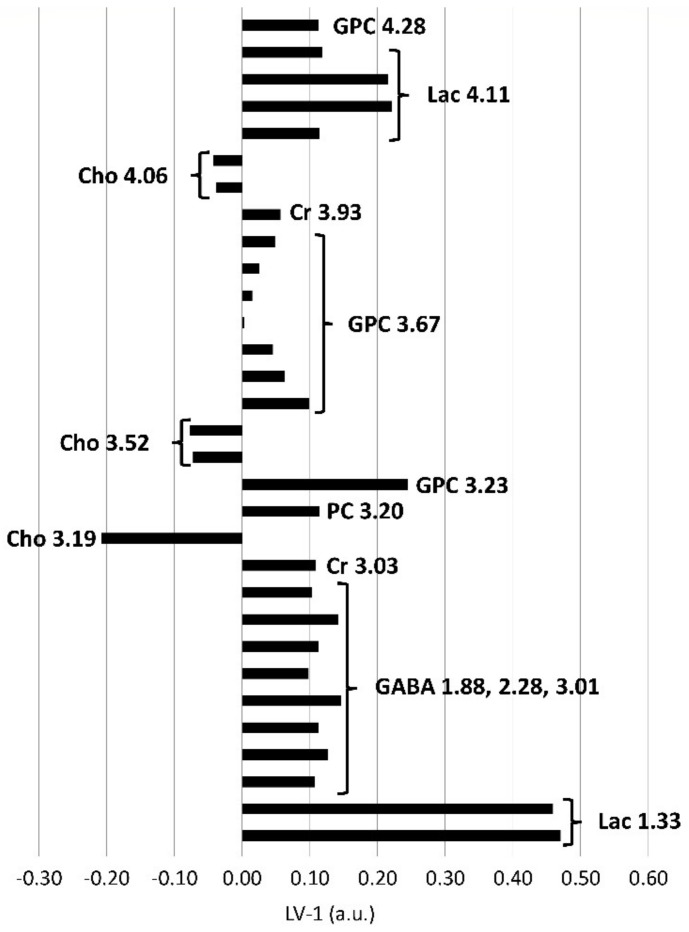
PLS-DA loadings of the first PLS-DA component (LV-1, arbitrary units) of thalamus biopsies demonstrating significant differences between in vivo and postmortem biopsies. Positive loadings correspond with metabolites present in increased concentrations, negative loadings with decreased concentrations in the in vivo biopsies compared to the postmortem biopsies. The length of the bar is positively correlated with its importance as a discriminator between groups in the PLS-DA model. Excerpt of buckets assigned to metabolites and their center frequencies in ppm beyond an arbitrary threshold of a PLS-DA coefficient of +/−0.1, below or above which they were considered as important discriminators. Additionally, corresponding resonances from the same metabolites including resonances below +/−0.1 are also shown. Cho, choline; Cr, creatine; GABA, ƴ-aminobutyric acid; GPC, glycerophosphocholine; PC, phospchocholine; Lac, lactate.

In the postmortem-harvested thalamus biopsies, the Cho peaks were lower, and the Lac, Cr, PC and GPC peaks were higher, compared with the in vivo-harvested biopsies. In addition, GABA was increased in the postmortem compared with the in vivo-harvested biopsies, as shown in Figure 6.

## 3. Discussion

We observed multiple metabolite alterations in the HR-MAS spectra of brain tissue samples obtained by postmortem surgical dissection (“postmortem-harvested samples”) compared to brain biopsies obtained by stereotactic biopsy under general anesthesia (“in vivo-harvested biopsies”). These alterations most likely reflect different stages of tissue degradation with respect to the period of sample ischemia. The in vivo-harvested biopsies collected using the minimally invasive stereotactic method showed fewer signs of decomposition than the postmortem-harvested samples, and are likely to be closer to the situation in the living animal.

### 3.1. Regional Differences in the Metabolic Profile

In accordance with previous studies [7,13], we observed relatively increased concentrations of NAAG and mIns in the brainstem compared to the thalamus, and NAA and PC in the thalamus compared to the brainstem in both in vivo- and postmortem-harvested samples, underlining the importance of the differences in the metabolic profiles of brain regions.

### 3.2. Increased Lactate as a Sign for the Switch of Energy Metabolism to Anaerobic Glycolysis

Increased concentrations of lactate occur rapidly in pathological conditions, such as hypoxia and ischemia, associated with a switch to anaerobic glycolysis [1,14,15].

Lactate is generally not observed in in vivo MRS studies of the brain, as it exists in very low concentrations in the normal brain [14]. It is present in both in vivo- and postmortem-harvested samples in high concentrations. It is the highest peak in the spectrum of the postmortem-harvested samples and is relatively increased in the postmortem- compared to the in vivo-harvested samples. In postmortem MRS studies of sheep and human brains, lactate appears within the first hours after death; however, it does not represent the highest peak in the spectrum [16,17]. The pronounced rise in lactate in both of our sample groups is consistent with anaerobic glycolysis due to sample ischemia, and is in agreement with the HR-MAS results of Opstad et al. [6], showing an increase in lactate concentration in samples of rat cerebral cortex within 30 min of ischemia, but no further increase up to 3 h. Shank et al. [10] showed an increase in lactate concentration in the rat brain within the first 2 to 4 min after decapitation, which did not increase further within 10 min. We observed an increase in lactate in both sample groups, with the lactate concentration being relatively higher in the postmortem-harvested samples (snap frozen no later than 20 min after death) than in the in vivo-harvested biopsies (snap frozen no later than 3 min after sampling), and with both groups being examined by an HR-MAS experiment over 1 h at a nominal temperature of 278 K. This underlines the rapid increase in lactate in the brain tissue samples within minutes of ischemia, but also indicates that anaerobic glycolysis is ongoing at least between 3 and 20 min of ischemia in goat brain.

### 3.3. Creatine as a Marker of Impaired Brain Energy Homeostasis

Creatine acts as an organic osmolyte in the brain [18]. It is also implicated in the arginine and proline metabolism and in the glycine and serine metabolism [19], and plays a pivotal role in brain energy homeostasis [18].

In contrast to studies of postmortem metabolite levels in rat brain [11] and bovine brain homogenate [20], where creatine levels were constant, we observed a relative increase in creatine in the postmortem-harvested biopsies compared to the in vivo-harvested biopsies. The breakdown of phosphocreatine during sampling or the NMR experiment may be a possible source of the increase in creatine in the postmortem samples. However, we did not observe a decrease in phosphocreatine accompanying the rise in creatine in the postmortem-harvested samples compared to the in vivo-harvested biopsies. Opstad et al. [6] reported a rise in the concentration of creatine in rat brain cortex over time in a continuous spinning experiment, but not in ischemic brain samples, which underwent only a short HR-MAS experiment. The increase in creatine concentration during continuous spinning was interpreted as a release of NMR-invisible bound creatine resulting from tissue damage due to magic angle spinning. However, it seems questionable that this explains the creatine increase in our study, since the NMR part in our experiment was identical for the in vivo and postmortem samples, with the sampling method and delay in initial sample freezing being the only difference. In addition, the mechanical stress for the in vivo group due to the negative pressure method of in vivo biopsy was even higher than for the postmortem-harvested samples, which were cut with the scalpel. Our data suggest that time and temperature factors are also important, and that possibly brain region (cortex vs. brainstem and thalamus) and species (rat vs. goat) may have an additional influence.

### 3.4. Increase of Acetate and ƴ-Aminobutyric Acid (GABA) May Reflect the Breakdown of Lipids and Myelin

Acetate is used for the synthesis of myelin lipids [18], is involved in the metabolism of aspartate, which is an important excitatory neurotransmitter, and is at the same time also part of the pyruvate metabolism, which is an important metabolite for anaerobic glycolysis [19].

In accordance with the data of Opstad et al. [6] and Peeling et al. [9], who both reported a strong increase in acetate but no changes in *N*-acetylaspartate (NAA) or aspartate in the rat brain intact tissue biopsies and in perchloric acid tissue extracts, respectively, we also saw a relative increase in acetate concentration in postmortem- compared to in vivo-harvested brainstem biopsies, but no corresponding changes in NAA or in aspartate. This increased acetate level may be partly due to the metabolic degradation of fatty acids released by membrane degradation and turnover, as suggested by Peeling et al. [9].

ƴ-aminobutyric acid (GABA) is considered as the main inhibitory neurotransmitter in the vertebrate central nervous system [19]. Several studies observed a postmortem cellular release and increase in GABA in the rat hippocampus [8], cortex [6] and brain [12]. Interestingly, in the analysis of the brain regions of the brainstem and thalamus, we observed a relatively decreased GABA concentration in the thalamus compared to the brainstem in the in vivo-harvested biopsies, and a relatively increased concentration in the thalamus compared to the brainstem in the postmortem-harvested samples. This may indicate that the postmortem increase in the thalamus is much stronger than in the brainstem. This finding might reflect possible regional differences in GABA concentrations similar to the regional differences in the localization of GABA receptors [21].

### 3.5. Alterations in the Choline Group and Increased Concentrations of Inositol Sugars Suggest Membrane Damage

Choline is required for the synthesis of the neurotransmitter acetylcholine and is a precursor of important components of biological membranes [14]. Changes in the concentrations of choline-containing metabolites (choline, phosphocholine and glycerophosphocholine) have been implicated in both cell proliferation and death processes [22], and are generally associated with alterations of membrane composition [14] (either increased membrane synthesis or breakdown) or changes in cell density [18].

In biopsies of both brainstem and thalamus, a relative decrease in choline and a relative increase in phosphocholine and glycerophosphocholine was seen in the samples harvested postmortem compared to the samples harvested in vivo by stereotactic biopsy. The increase in phosphocholine and glycerophosphocholine reflects most likely the degradation of membranes [6,18]. However, the underlying mechanism of the observed decrease in choline remains unclear, as usually situations that impair energy supply in the brain result in high brain levels of free choline [18].

*Myo*-inositol and *scyllo*-inositol are stereoisomers of the sugar alcohol inositol [14]. *Myo*-inositol plays a role as a constituent of phosphoglyceride, a lipid component of bio-membranes, acts as an osmolyte, and is a precursor molecule for components in the intracellular second messenger system [18,19].

Tsang et al. [7] applied HR-MAS NMR to characterize different brain regions in rats, and reported the increase in myo-inositol as one of the most important metabolites to distinguish samples of the brainstem from samples of other brain regions. We also observed relatively higher myo-inositol concentrations in the brainstem compared to the thalamus in both the in vivo- and postmortem-harvested samples. Furthermore, we observed a relative increase in the concentrations of myo-inositol and scyllo-inositol in the postmortem- compared to the in vivo-harvested samples of the brainstem, but not in the thalamus. The release of myo-inositol from brain cells might serve to counteract cell swelling [23], and may be compatible with membrane damage and decomposition processes.

### 3.6. Limitations

Since we took more than one biopsy from the same site at a minor time interval, an influence of previous sampling on in vivo and postmortem samples cannot be excluded. We used a different technique for in vivo and postmortem sampling (stereotactic biopsy using a passive side cutting needle versus dissection using a scalpel, respectively), which exposes the tissue to different forces. Nevertheless, this reflects clinical reality, as it is common practice in human medicine to take more than one sample using different techniques.

We used snap frozen unprocessed tissue for the HR-MAS experiments in order to not introduce metabolic changes by tissue fixation, for example, by sample heating; however this may have led to ongoing metabolic processes during the HR-MAS experiment. We chose an HR-MAS experiment with a fixed duration of one hour at 278 K based on the desired signal-to-noise ratio, and a quick and reliable start of the acquisition after the insertion of the rotor, which also may have influenced the ongoing metabolic processes. However, the timing protocol used was kept identical for all samples.

Whether pentobarbitalum, which we used for the euthanasia of our goats, has an effect on the metabolic spectra is under discussion. Jernerén et al. [24] described a significant influence on the content of free fatty acids in the brain depending on the method of euthanasia. We used a sequence with a T2 filter reducing the signal intensity of fatty acids in the spectra, and therefore minimized the effect of fatty acids in our data analysis. We cannot totally rule out the effect of pentobarbitalum, but we consider the metabolic processes that take place in the period after death and before sampling to be more decisive.

## 4. Materials and Methods

### 4.1. Brain Biopsies and Sampling Methods

A total of 32 biopsies was obtained; from each of the 4 healthy goats a total of 8 samples was collected. Each goat was sampled at 4 different sites (brain stem left and right, thalamus left and right). From each localization, one in vivo and one postmortem sample was harvested. In vivo biopsies were taken by minimally invasive stereotactic brain biopsy under general anesthesia [25]. After advancing the biopsy needle (passive 16-gauge side-cutting biopsy needle, Rogue Research Inc., Canada) to the target, the cutting window was opened, suction was drawn using a syringe attached to the inner cannula hub for 30 s, the biopsy window was closed and the inner cannula was withdrawn. The biopsy was flushed from the biopsy needle using sterile saline. Within 3 min after the insertion of the biopsy needle the biopsy was snap frozen in liquid nitrogen and stored at −80 °C. Up to 4 biopsies were taken at each target by turning the needle clockwise and obtaining a biopsy at positions 0°, 90°, 180° and 240°. As the goats served as the control group in another study [13], only one of the biopsies was used for this study. The goats were euthanized immediately after in vivo biopsy using pentobarbitalum (Euthasol ^®^, Virbac AG, Switzerland, dosage: 400 mg/kg intravenously). Brains were rapidly removed after death and postmortem samples were taken by cutting the brain manually with a scalpel. Samples were collected from tissue adjacent to the area from which the in vivo-harvested biopsies were obtained. All postmortem-harvested samples were snap frozen in liquid nitrogen within 20 min of death and stored at −80 °C. The entire procedure and timing were kept as much as possible identical for all biopsies of both sampling procedures (in vivo biopsies 3 min, postmortem biopsies 20 min). All experiments were carried out according to local ethics regulations (Swiss Veterinary Service, Office of Agriculture and Nature, approval number BE 56/10). The absence of central nervous system (CNS) disease in the brains of the animals was confirmed by histopathology.

### 4.2. ^1^H High-Resolution–Magic Angle Spinning (HR-MAS) NMR Spectroscopy

Each biopsy was thawed and weighted; postmortem-harvested samples were cut if they were too large (>15 mg) and then each biopsy was placed in a zirconium oxide rotor with a 12 µl insert. The remaining space was filled with D_2_O-based phosphate buffered saline (PBS, 50 mM, pH 7). In order to minimize the possible metabolic changes caused by the metabolic degradation processes, each sample was taken out of the −80 °C freezer immediately before preparing the rotor for the HR-MAS experiment. In addition, care was taken to ensure that the time between taking the sample out of the freezer and the start of the first scan at a stable temperature of 278 K remained as identical as possible for all samples (in vivo samples: 36 +/− 8 min; postmortem samples: 45 +/− 11 min). All experiments were performed in a 500.13 MHz Bruker Avance II spectrometer (Bruker Biospin AG, Fällanden, Switzerland) equipped with a 4 mm HR-MAS dual inverse (^1^H, ^13^C) probe. Spectra were acquired in a randomized order using a 1D ^1^H sequence with water presaturation and with a T2-filter eliminating J-modulation (“PROJECT” Periodic Refocusing of J Evolution by Coherence Transfer [26]) at a 3 kHz MAS rate and nominal 278 K. Each 1D ^1^H NMR spectrum was acquired by applying 512 transients, a spectral width of 6010 Hz, a data size of 32 K points, an acquisition time of 2.73 s, a relaxation delay of 4 s, an echo time (TE) of 400 ms, and a rotor-synchronized interpulse delay of 1.33 ms [27]. After testing several TEs, a TE of 400ms was chosen to achieve an optimal balance between a sufficient signal of the small metabolite peaks and the suppression of broad components from macromolecules and lipids, which otherwise may have introduced bias into the integration of the small metabolite peaks. To support the ^1^H NMR resonance assignments, 2D ^1^H^1^H-TOCSY spectra using the DIPSI2 sequence for mixing (“dipsi2phpr” from the Bruker pulse program library) with presaturation during relaxation delay were recorded for selected samples, with a spectral width of 5000 Hz, acquisition time of 205 ms, 96 scans per increment and 256 increments (Figure S5).

### 4.3. Data Analysis

The post-processing of spectra included Fourier transformation, manual phasing and chemical shift calibration to the resonance of creatine (Cr) at 3.03 ppm. After the post-processing of spectra using TopSpin (3.5, Bruker Biospin GmbH), spectral assignments were performed using literature references [14], additionally performed 2D measurements and data obtained from the Human Metabolome Database (http://www.hmdb.ca, version 4.0, 2018) [19]. Chemometric analysis to determine metabolic differences between the two biopsy types was performed using a home-written Matlab script (R2011b, The MathsWorks Inc., Natick, MA, USA) and the PLS toolbox (Eigenvector Research Inc., Manson, Washington, WA, USA). For multivariate analysis using unsupervised principal component analysis (PCA) and partial least squares discriminant analysis (PLS-DA), 250 buckets of variable sizes according to peak width were defined between 0.9 and 8.9 ppm after exclusion of areas of pure noise, lipids and the contaminant ethanol [14]. The average bucket width was 0.014 ppm (minimum 0.003 ppm; maximum, 0.076 ppm; SD 0.01). Table S1 in the supplement contains a list of the buckets used. The bucket integrals were normalized by probabilistic quotient normalization (PQN). All integrals were mean-centered and Pareto-scaled.

To investigate the differences between the two sampling types, unsupervised PCA was applied to test for clustering. The scores of the first principal component were tested for differences between the groups using the Wilcoxon test (*p* < 0.05) in Excel (Office 2011, Microsoft Corporation, Redmond, WA, USA). Then, PLS-DA was applied to test for the separation of the groups. PLS-DA models were subsequently subjected to permutation testing (cross-validation, 50 iterations). To interpret loading values, any beyond an arbitrary threshold of +/−0.1 were considered as strong contributors. After assigning resonances, the multiple resonances of a specific metabolite were analyzed and the metabolite was only assumed to discriminate between groups if all resonances showed a consistent pattern.

In addition, PLS-DA was applied to test for differences between samples obtained from the left or right side for both sampling types and both brain regions. Samples of both sampling methods were also tested for differences between the brainstem and the thalamus.

## 5. Conclusions

In conclusion, we showed that taking brain biopsies in vivo under general anesthesia using a minimally invasive stereotactic method is associated with different metabolic alterations reflecting less tissue degradation compared with biopsies that were taken surgically postmortem with a time delay of maximal 20 min after death. Lactate, creatine and choline-containing compounds were altered most likely due to a switch to anaerobic glycolysis and membrane breakdown. In addition, we also observed similarities, but also differences, in the metabolic changes between in vivo- and postmortem-harvested samples, when collected from the brainstem and the thalamus, respectively. Acetate and inositols were relatively increased in the postmortem compared to the in vivo samples of the brainstem, as was ƴ-aminobutyric acid in the thalamus, demonstrating regional differences in tissue degradation. Consequently, these metabolic alterations have to be taken into account when examining samples obtained by different sampling methods and from different brain regions. To be as close as possible to the actual situation in the living individual, it is desirable to use brain samples obtained by stereotactic biopsy whenever possible.

## Data Availability

The data presented in this study are available on request from the corresponding author.

## Supplementary Materials

The following are available online at https://www.mdpi.com/2218-1989/11/1/38/s1, Figure S1: PCA scores plots of in vivo (indicated by i) (a) and postmortem (indicated by p) (b) samples of the brainstem (B) and thalamus (T) of the left (L) and right side (R) showing no clustering of the samples obtained from an individual animal (1 to 4) in the in vivo (i) samples and clustering in half of the samples obtained from an individual animal in the postmortem (p) samples, Figure S2: PLS-DA scores plots of in vivo (i) (a) and postmortem (p) (c) samples of the brainstem (B) and in vivo (b) and postmortem (d) samples of the thalamus (T) showing a clustering of the samples obtained from an individual animal (1 to 4) from the left (L) and right (R) side, Figure S3: PLS-DA scores (a) and loadings (b) plots of in vivo biopsies of the brainstem and thalamus showing a clear separation between the brain regions and highlighting important discriminating metabolites beyond an arbitrary threshold of +/−0.1. Cho, choline; Cr, creatine; GABA, ɣ-aminobutyric acid; GSH, glutathione; Glu, glutamate; GPC, glycerophosphocholine; GSH, glutathione; Lac, lactate; *m*Ins, *myo*-inositol; NAA, *N*-acetylaspartate; NAAG, N-acetylaspartylglutamate; PC, phosphocholine; Pro, proline; Tau, taurin, Figure S4: PLS-DA scores (a) and loadings (b) plots of postmortem biopsies of the brainstem and thalamus showing a clear separation between the brain regions and highlighting important discriminating metabolites beyond an arbitrary threshold of +/−0.1. GABA, ɣ-aminobutyric acid; Gln, glutamine; Glu, glutamate; Lac, lactate; *m*Ins, *myo*-inositol; NAA, *N*-acetylaspartate; NAAG, N-acetylaspartylglutamate; PC, phosphocholine; Tau, taurine, Figure S5: 2D ^1^H^1^H-TOCSY spectrum of a postmortem sample from the left thalamus of goat 2. Ala, alanine; Asp, aspartate; Arg, Arginine; Cho, choline; EtOH, ethanol; GABA, ɣ-aminobutyric acid; Gln, glutamine; GSH, glutathione; Glu, glutamate; GPC, glycerophosphocholine; Lac, lactate; Leu, Leucine; Lys, Lysine; Ile, Isoleucine; *m*Ins, *myo*-inositol; NAA, *N*-acetylaspartate; NAAG, N-acetylaspartylglutamate; PC, phosphocholine; Ser, Serine; Tau, taurine. Table S1: List of buckets including spectral regions.
