# Supplementary material for: Sampling Method Affects HR-MAS NMR Spectra of Healthy Caprine Brain Biopsies
Source: Metabolites. 2021 Jan 6;11(1):38. doi: 10.3390/metabo11010038 (PMC7825498; doi:10.3390/metabo11010038)
Supplement: Supplementary file 1 [file metabolites-11-00038-s001.pdf]

Supplementary material:

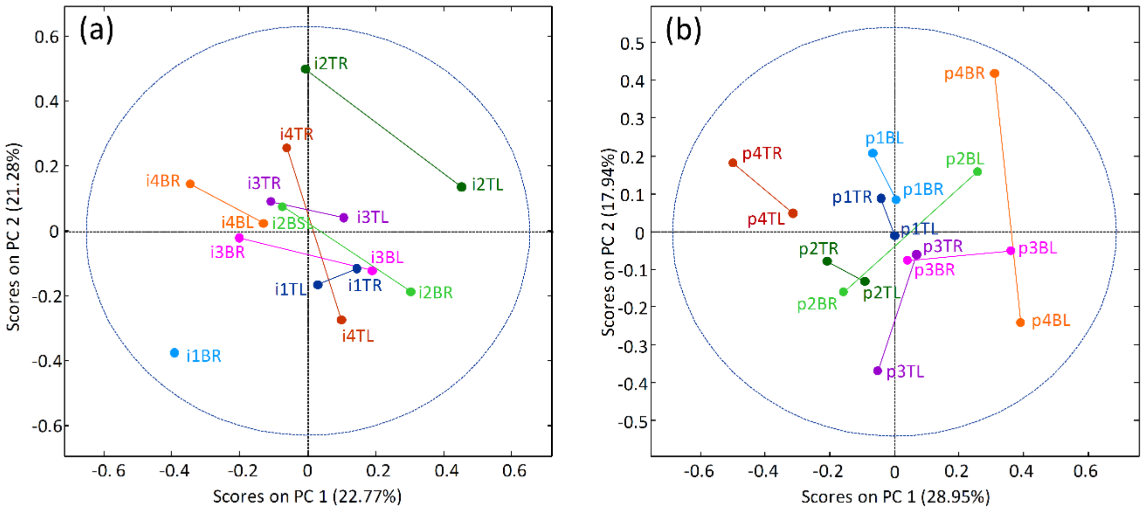

Figure S1: PCA scores plots of *in vivo* (indicated by i) (a) and post mortem (indicated by p) (b) samples of the brainstem (B) and thalamus (T) of the left (L) and right side (R) showing no clustering of the samples obtained from an individual animal (1 to 4) in the *in vivo* (i) samples and clustering in half of the samples obtained from an individual animal in the post mortem (p) samples

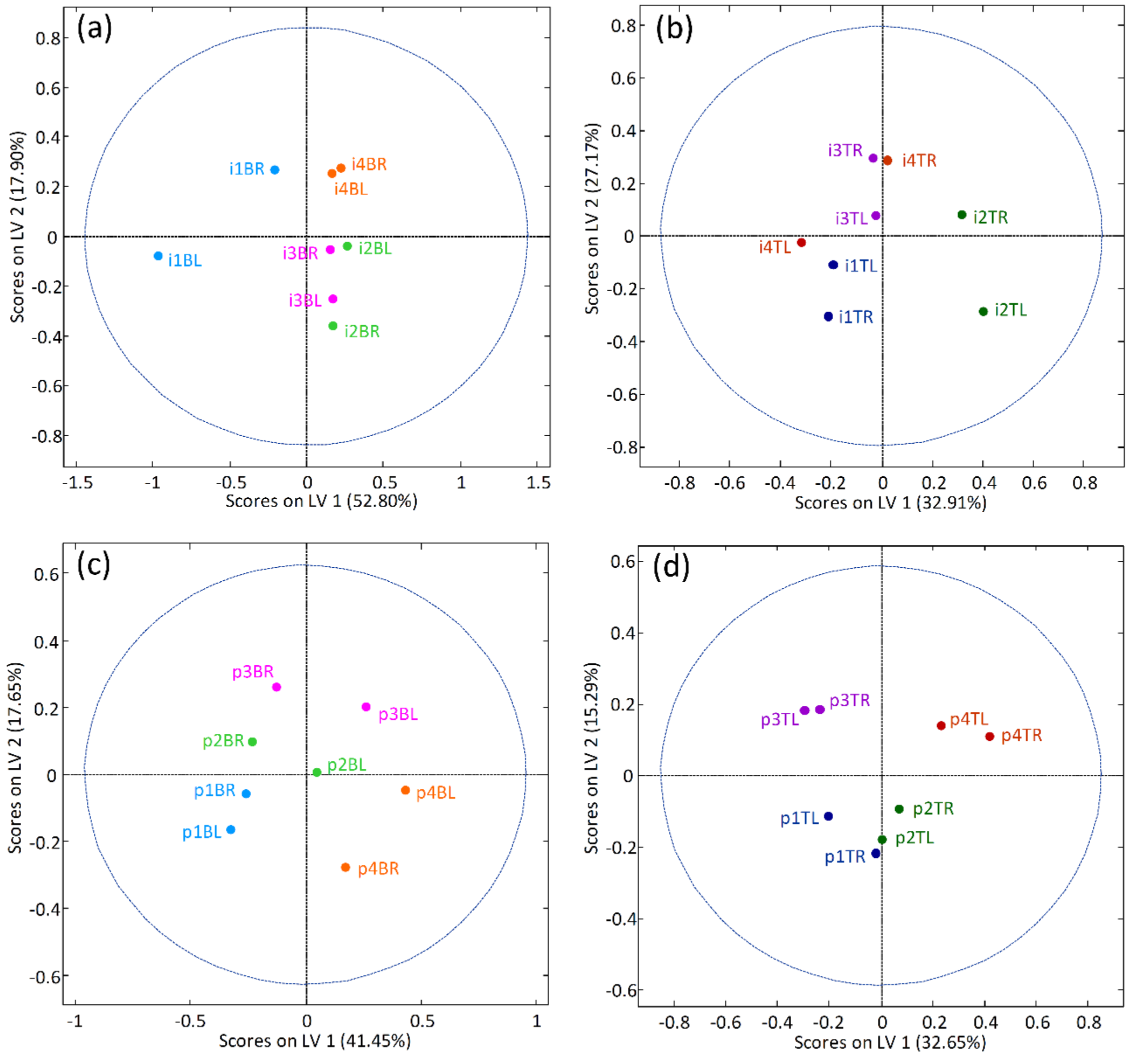

Figure S2: PLS-DA scores plots of *in vivo* (i) (a) and post mortem (p) (c) samples of the brainstem (B) and *in vivo* (b) and post mortem (d) samples of the thalamus (T) showing a clustering of the samples obtained from an individual animal (1 to 4) from the left (L) and right (R) side

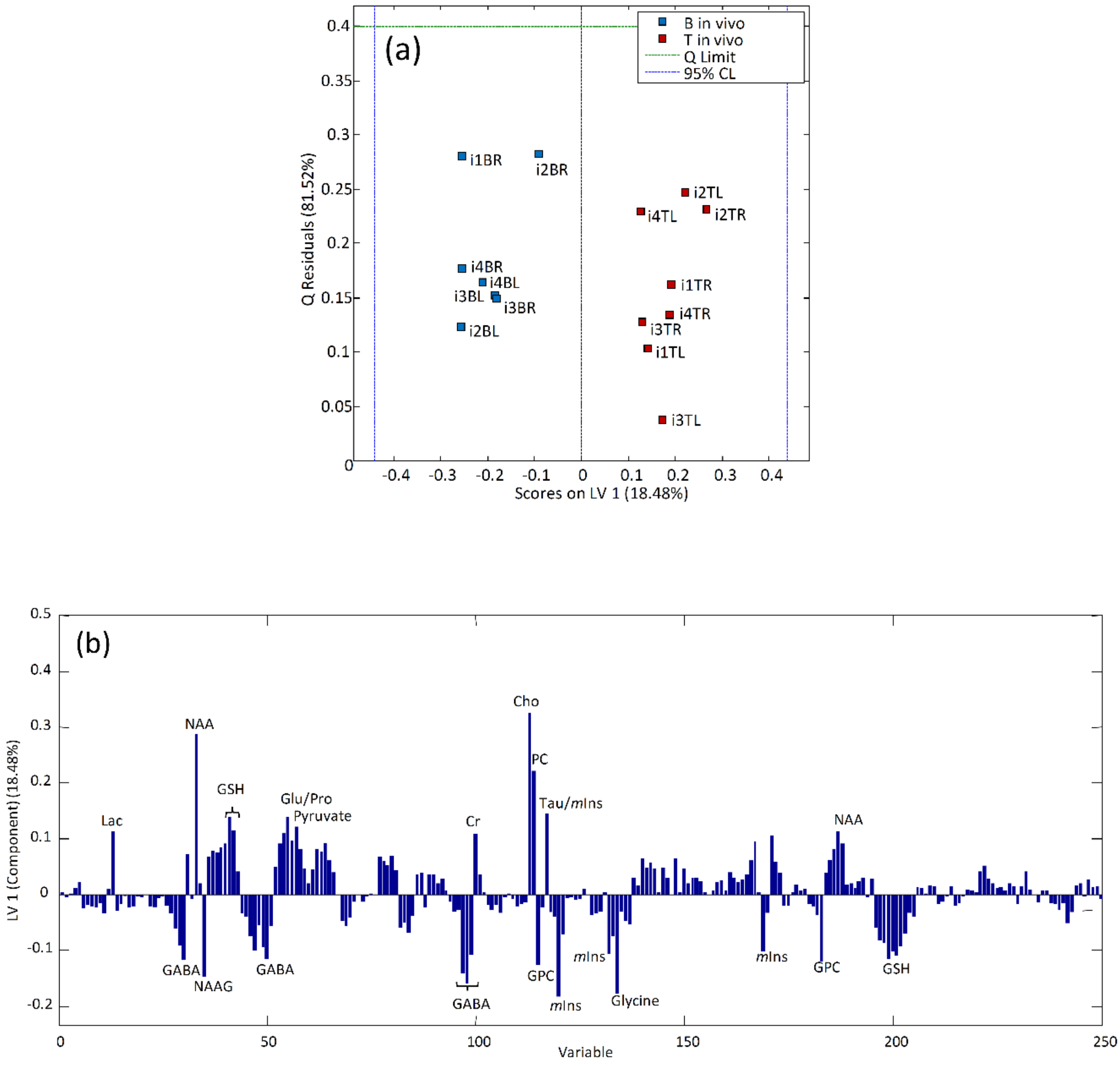

Figure S3: PLS-DA scores (a) and loadings (b) plots of *in vivo* biopsies of the brainstem and thalamus showing a clear separation between the brain regions and highlighting important discriminating metabolites beyond an arbitrary threshold of +/- 0.1. Cho, choline; Cr, creatine; GABA, ɣ-aminobutyric acid; GSH, glutathione; Glu, glutamate; GPC, glycerophosphocholine; GSH, glutathione; Lac, lactate; *m*Ins, *myo*-inositol; NAA, *N*-acetylaspartate; NAAG, N-acetylaspartylglutamate; PC, phosphocholine; Pro, proline; Tau, taurin

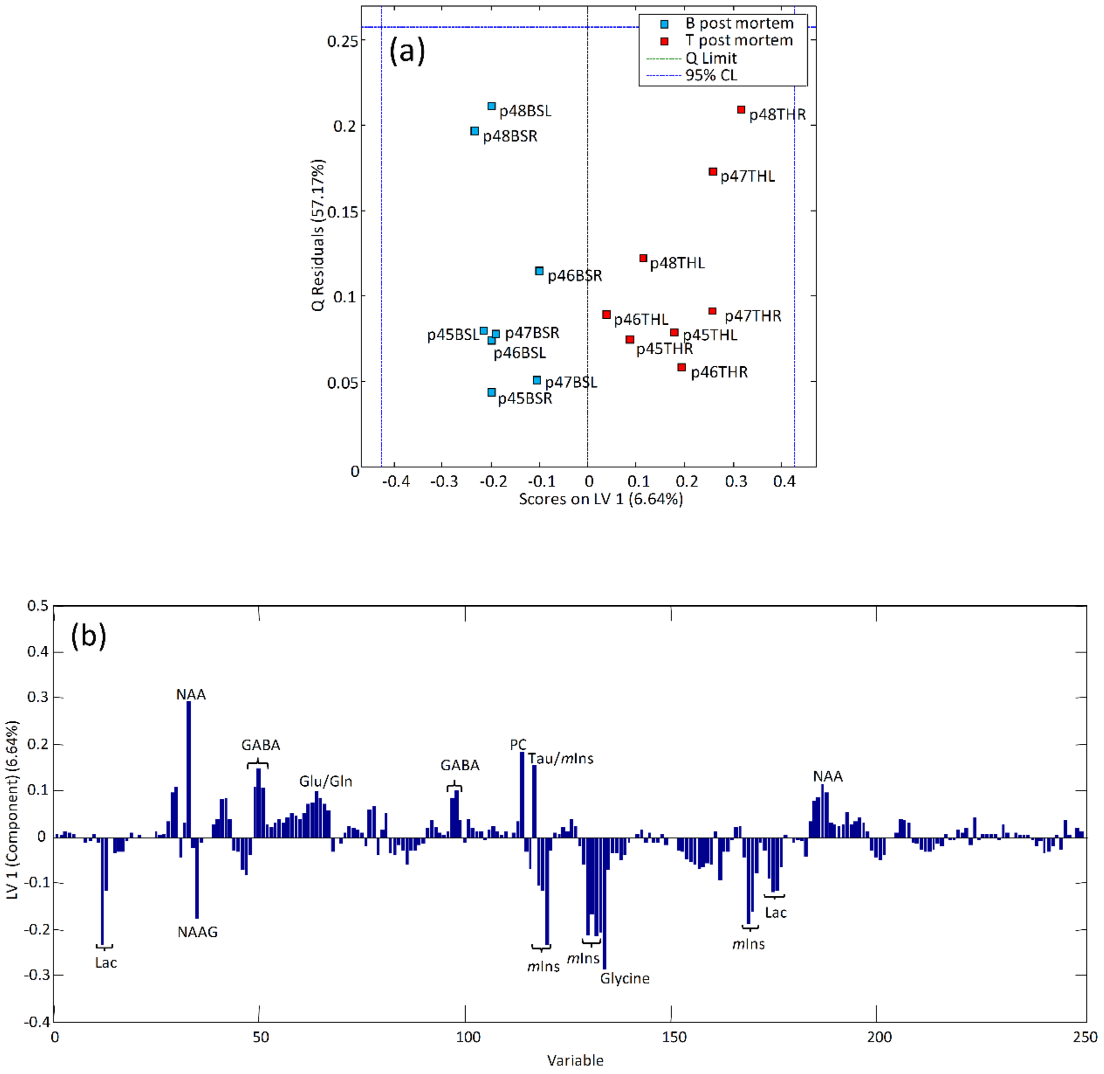

Figure S4: PLS-DA scores (a) and loadings (b) plots of post mortem biopsies of the brainstem and thalamus showing a clear separation between the brain regions and highlighting important discriminating metabolites beyond an arbitrary threshold of +/- 0.1. GABA, ɣ-aminobutyric acid; Gln, glutamine; Glu, glutamate; Lac, lactate; *m*Ins, *myo*-inositol; NAA, *N*-acetylaspartate; NAAG, N-acetylaspartylglutamate; PC, phosphocholine; Tau, taurine

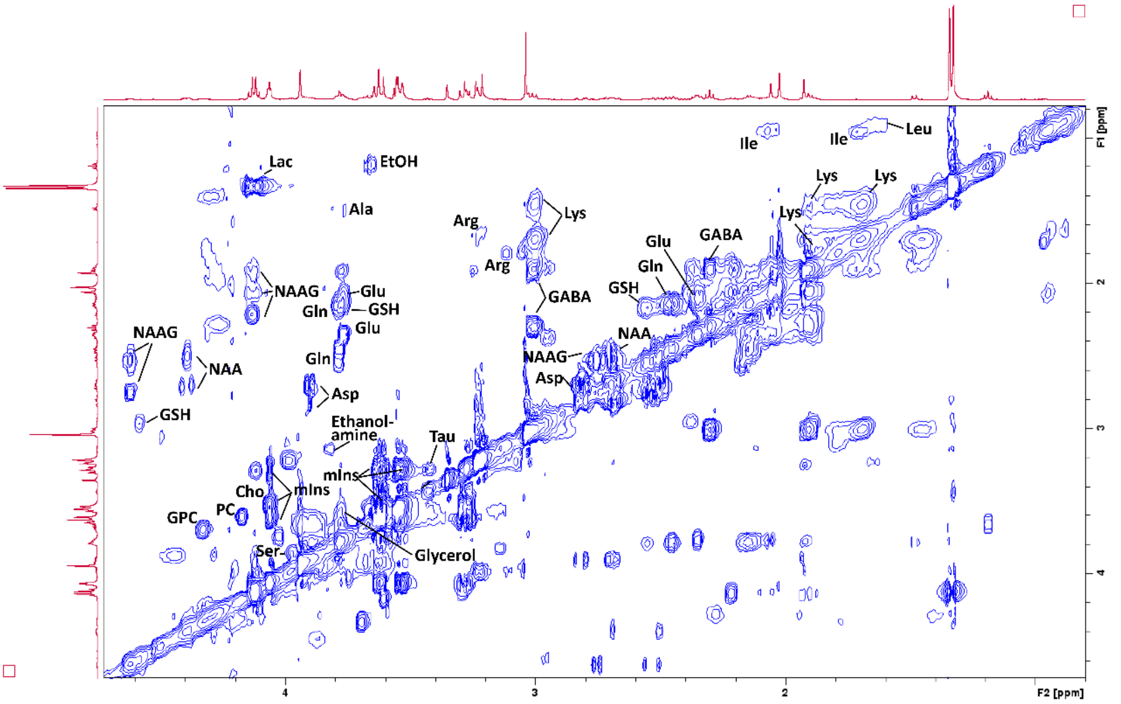

Figure S5: 2D ^{1}H^{1}H-TOCSY spectrum of a post mortem sample from the left thalamus of goat 2**.** Ala, alanine; Asp, aspartate; Arg, Arginine; Cho, choline; EtOH, ethanol; GABA, ɣ-aminobutyric acid; Gln, glutamine; GSH, glutathione; Glu, glutamate; GPC, glycerophosphocholine; Lac, lactate; Leu, Leucine; Lys, Lysine; Ile, Isoleucine; *m*Ins, *myo*-inositol; NAA, *N*-acetylaspartate; NAAG, N-acetylaspartylglutamate; PC, phosphocholine; Ser, Serine; Tau, taurine.

Table S1. List of buckets including spectral regions

Bucket number	Bucket right limit (ppm)	Bucket left limit (ppm)	Bucket size (ppm)
1	0.916	0.929	0.013
2	0.929	0.941	0.012
3	0.941	0.953	0.012
4	0.953	0.965	0.012
5	0.965	0.974	0.009
6	0.974	0.986	0.012
7	0.986	0.997	0.011
8	0.997	1.008	0.011
9	1.008	1.023	0.015
10	1.023	1.040	0.017
11	1.040	1.057	0.017
12	1.251	1.327	0.076
13	1.327	1.369	0.042
14	1.369	1.385	0.016
15	1.440	1.453	0.013
16	1.453	1.461	0.008
17	1.461	1.476	0.015
18	1.476	1.499	0.023
19	1.637	1.649	0.012
20	1.649	1.663	0.014
21	1.663	1.676	0.013
22	1.676	1.689	0.013
23	1.689	1.699	0.010
24	1.699	1.716	0.017
25	1.716	1.733	0.017
26	1.733	1.746	0.013
27	1.784	1.796	0.012
28	1.833	1.875	0.042
29	1.875	1.891	0.016
30	1.891	1.907	0.016
31	1.907	1.927	0.020
32	1.927	1.943	0.016
33	1.974	2.028	0.054
34	2.028	2.039	0.011
35	2.039	2.057	0.018
36	2.057	2.071	0.014
37	2.071	2.085	0.014
38	2.085	2.097	0.012
39	2.097	2.110	0.013
40	2.110	2.120	0.010

41	2.120	2.136	0.016
42	2.136	2.152	0.016
43	2.152	2.165	0.013
44	2.165	2.180	0.015
45	2.180	2.188	0.008
46	2.188	2.202	0.014
47	2.202	2.219	0.017
48	2.219	2.235	0.016
49	2.266	2.286	0.020
50	2.286	2.303	0.017
51	2.303	2.318	0.015
52	2.318	2.329	0.011
53	2.329	2.336	0.007
54	2.336	2.345	0.009
55	2.345	2.353	0.008
56	2.353	2.360	0.007
57	2.360	2.374	0.014
58	2.374	2.388	0.014
59	2.388	2.402	0.014
60	2.402	2.416	0.014
61	2.416	2.431	0.015
62	2.431	2.447	0.016
63	2.447	2.462	0.015
64	2.462	2.478	0.016
65	2.478	2.494	0.016
66	2.494	2.510	0.016
67	2.510	2.528	0.018
68	2.528	2.539	0.011
69	2.539	2.550	0.011
70	2.550	2.557	0.007
71	2.557	2.569	0.012
72	2.569	2.580	0.011
73	2.580	2.587	0.007
74	2.587	2.594	0.007
75	2.629	2.642	0.013
76	2.642	2.668	0.026
77	2.668	2.682	0.014
78	2.682	2.689	0.007
79	2.689	2.702	0.013
80	2.702	2.713	0.011
81	2.713	2.722	0.009
82	2.722	2.731	0.009
83	2.731	2.744	0.013
84	2.754	2.762	0.008
85	2.762	2.772	0.010
86	2.775	2.793	0.018
87	2.793	2.808	0.015

88	2.808	2.823	0.015
89	2.823	2.829	0.006
90	2.829	2.838	0.009
91	2.883	2.893	0.010
92	2.893	2.904	0.011
93	2.910	2.918	0.008
94	2.932	2.948	0.016
95	2.948	2.960	0.012
96	2.960	2.972	0.012
97	2.972	2.994	0.022
98	2.994	3.011	0.017
99	3.011	3.021	0.010
100	3.021	3.040	0.019
101	3.040	3.056	0.016
102	3.056	3.070	0.014
103	3.086	3.096	0.010
104	3.096	3.109	0.013
105	3.109	3.116	0.007
106	3.116	3.126	0.010
107	3.126	3.141	0.015
108	3.141	3.150	0.009
109	3.150	3.155	0.005
110	3.155	3.163	0.008
111	3.163	3.171	0.008
112	3.171	3.175	0.004
113	3.175	3.213	0.038
114	3.213	3.225	0.012
115	3.225	3.245	0.020
116	3.245	3.259	0.014
117	3.259	3.269	0.010
118	3.269	3.286	0.017
119	3.286	3.308	0.022
120	3.315	3.350	0.035
121	3.350	3.369	0.019
122	3.369	3.382	0.013
123	3.386	3.400	0.014
124	3.400	3.413	0.013
125	3.413	3.416	0.003
126	3.416	3.431	0.015
127	3.431	3.440	0.009
128	3.456	3.475	0.019
129	3.475	3.492	0.017
130	3.492	3.523	0.031
131	3.523	3.532	0.009
132	3.532	3.543	0.011
133	3.543	3.552	0.009
134	3.552	3.567	0.015

135	3.671	3.684	0.013
136	3.684	3.694	0.010
137	3.694	3.705	0.011
138	3.720	3.735	0.015
139	3.735	3.743	0.008
140	3.743	3.752	0.009
141	3.752	3.759	0.007
142	3.759	3.763	0.004
143	3.763	3.767	0.004
144	3.767	3.773	0.006
145	3.773	3.779	0.006
146	3.779	3.783	0.004
147	3.783	3.787	0.004
148	3.787	3.798	0.011
149	3.798	3.805	0.007
150	3.805	3.811	0.006
151	3.811	3.822	0.011
152	3.822	3.835	0.013
153	3.835	3.846	0.011
154	3.846	3.855	0.009
155	3.855	3.865	0.010
156	3.865	3.876	0.011
157	3.876	3.886	0.010
158	3.886	3.896	0.010
159	3.896	3.903	0.007
160	3.903	3.911	0.008
161	3.911	3.921	0.010
162	3.921	3.945	0.024
163	3.945	3.955	0.010
164	3.955	3.965	0.010
165	3.965	3.972	0.007
166	3.972	3.982	0.010
167	3.982	4.013	0.031
168	4.013	4.026	0.013
169	4.026	4.052	0.026
170	4.052	4.059	0.007
171	4.059	4.073	0.014
172	4.073	4.078	0.005
173	4.078	4.085	0.007
174	4.085	4.101	0.016
175	4.101	4.117	0.016
176	4.117	4.132	0.015
177	4.132	4.145	0.013
178	4.247	4.261	0.014
179	4.261	4.271	0.010
180	4.271	4.278	0.007
181	4.278	4.286	0.008

182	4.286	4.294	0.008
183	4.294	4.348	0.054
184	4.359	4.369	0.010
185	4.369	4.377	0.008
186	4.377	4.385	0.008
187	4.385	4.398	0.013
188	4.398	4.408	0.010
189	4.426	4.433	0.007
190	4.433	4.444	0.011
191	4.444	4.451	0.007
192	4.473	4.485	0.012
193	4.485	4.510	0.025
194	4.510	4.518	0.008
195	4.518	4.528	0.010
196	4.554	4.566	0.012
197	4.566	4.581	0.015
198	4.581	4.592	0.011
199	4.592	4.605	0.013
200	4.605	4.614	0.009
201	4.614	4.625	0.011
202	4.625	4.634	0.009
203	4.634	4.646	0.012
204	4.646	4.651	0.005
205	4.651	4.663	0.012
206	4.754	4.769	0.015
207	4.769	4.782	0.013
208	4.782	4.791	0.009
209	5.784	5.797	0.013
210	5.797	5.809	0.012
211	5.876	5.891	0.015
212	5.891	5.908	0.017
213	5.908	5.919	0.011
214	5.919	5.931	0.012
215	6.088	6.101	0.013
216	6.101	6.117	0.016
217	6.510	6.525	0.015
218	6.789	6.799	0.010
219	6.875	6.888	0.013
220	6.888	6.904	0.016
221	7.129	7.148	0.019
222	7.148	7.162	0.014
223	7.162	7.182	0.020
224	7.182	7.223	0.041
225	7.297	7.318	0.021
226	7.318	7.340	0.022
227	7.340	7.355	0.015
228	7.355	7.362	0.007

229	7.362	7.373	0.011
230	7.373	7.393	0.020
231	7.393	7.413	0.020
232	7.413	7.425	0.012
233	7.516	7.531	0.015
234	7.531	7.550	0.019
235	7.569	7.581	0.012
236	7.581	7.588	0.007
237	7.588	7.597	0.009
238	7.597	7.613	0.016
239	7.876	7.890	0.014
240	7.890	7.907	0.017
241	8.178	8.200	0.022
242	8.200	8.221	0.021
243	8.221	8.246	0.025
244	8.246	8.271	0.025
245	8.337	8.369	0.032
246	8.369	8.408	0.039
247	8.691	8.704	0.013
248	8.704	8.717	0.013
249	8.923	8.933	0.010
250	8.933	8.947	0.014
